# A comparative shape analysis of the cervical spine between individuals with cervicogenic headaches and asymptomatic controls

**DOI:** 10.1038/s41598-021-98981-y

**Published:** 2021-09-30

**Authors:** Youssef Masharawi, Aumayma Murad Mansour, Natan Peled, Asaf Weisman

**Affiliations:** 1 Spinal Research Laboratory, Department of Physical Therapy, Stanley Steyer School of Health Professions, Sackler Faculty of Medicine, Tel Aviv University, Tel Aviv, Israel; 2 Department of Radiology, Carmel Medical Center, Haifa, Israel

**Keywords:** Anatomy, Medical research, Neurology

## Abstract

As some researchers theorized that cervicogenic headache (CEH) might be related to bony and discal features of the cervical spine, this retrospective study examined the shapes of the cervical vertebrae and intervertebral discs (IVDs) of individuals with CEH and compared them to asymptomatic controls. Scans of 40 subjects in their late 20’s–mid 30’s affected with CEH and 40 asymptomatic controls were obtained (overall = 19,040 measurements, age-sex matched, 20 males and 20 females in each group). The following cervical spine variables were measured: Supine lordosis, vertebral body-heights, A-P lengths, mediolateral widths and sagittal-wedging; IVDs heights and sagittal-wedging; pedicle heights, widths and transverse angles; laminar widths and transverse angles; articular facet angles, spinal canal, and transverse foramen lengths, widths, and areas. Both groups had similar shape variation along the cervical in all the measured parameters. There were no significant left–right differences in all measured parameters and no significant differences between the CEH and control groups concerning sex and age. Cervical IVDs were lordotic in shape, whereas their adjacent vertebral bodies were kyphotic in shape except for C2. In conclusion, the shape of the cervical spine and IVDs in subjects in their late 20’s–mid 30’s affected with CEH is identical to asymptomatic controls.

## Introduction

Cervicogenic headache (CEH) is considered a unique type of headache^1,2^. However, its diagnosis is based mainly on inconclusive signs and the temporality of subjective symptoms, including unilateral head pain, reduced neck range of motion, ipsilateral arm discomfort, a paroxysm of pain, and various attack-related events^3–5^. Conditions such as migraines and tension headaches further complicate the clinical diagnosis of CEH due to an overlap of symptoms^6^. An integral part of the diagnostic procedures of CEH relies on local anesthetic blocks, mainly of the facet joints^7^. While this approach is considered the “gold standard” by some practitioners, it remains a risky and challenging procedure as there could be other affected nociceptive generating structures in a patient^6,7^.

Some research suggests certain cervical anatomical variations (e.g., reduced intervertebral disc heights), functional abnormalities (e.g., segmental instability^8^), and a multitude of other common cervical findings (e.g., uncovertebral arthrosis, osteophytes, disc protrusions, fatty infiltration in cervical muscles, reduced cross-sectional areas of cervical muscles, narrowed subarachnoid space, and ventral dura compression)^9–16^ can contribute to the pathophysiology of CEH^2,7,17–19^. Considering this, CEH is a secondary manifestation of nociceptive inputs emanating from sensory afferent nerves that converge in the cervico-trigeminal nucleus at the dorsal horn of the C1-C3 levels^18,20^. Notably, these sensory afferent nerves have collateral branches that may ascend or descend for up to three segments before synapsing in the dorsal horn^21,22^. Consequently, all cervical segments may also be involved in the development of CEH^15^.

The International Classification of Headaches (ICHD 3)^5^ stated, however, that cervical findings in CEH as indicated in imaging (CT and MRI), should only be considered suggestive. It is therefore reasonable to consider them as just one element of a more extensive neurophysiological explanation for CEH. This includes a central predisposition to headaches and a tendency to a reduced headache threshold^5,15^. Accordingly, CEH could be triggered by a cervical component interacting with a central component. Moreover, patients predisposed to migraines and paroxysmal hemicrania could experience an attack triggered by nociceptive input originating from the neck (i.e., “cervicogenic migraine”)^15^.

Those suggested neurophysiological interactions highlight the need to include peripheral and central mechanisms when dealing with CEH, and emphasize the importance of confirming the non-contribution of the cervical anatomical structures to CEH as hypothesized in the current study.

### Objectives

This study aims to confirm the non-contribution of the morphology of vertebral and discal elements along the entire cervical spine to CEH, and compare it to asymptomatic controls.

## Results

We evaluated the CT scans of 80 cervical spines performed between 2009 and 2016 (40 CEH, 40 control/20 males, 20 females in each group), and there were no missing data. Both the intra-tester and inter-tester reliability for all measurements were high (inter-tester reliability 0.85 < ICC_3, 1_ < 0.95, intra-tester 0.72 < ICC_3, 1_ < 1.00). All measured variables were normally distributed in the two groups (Kolmogorov–Smirnov: 0.26 < p < 0.91). The ANOVA analysis with a Welch and post hoc test and Bonferroni correction revealed no significant differences in all measured independent variables except for individual’s height (Table 1) and dependent variables between the CEH and controls (0.57 < p < 0.87). In both groups, females were significantly shorter than males (p = 0.003) (Table 1). All measured vertebral and IVDs measurements in both groups were greater in males than in females (ANOVA, p < 0.05). Supine cervical lordosis measured from C2 to C7 was similar in all individuals, ranging between 32.2° and 36° (1.2 < SD < 1.60). There were no significant left–right differences in all measured parameters in the two studied groups, CEH, and controls (Paired t-test, p > 0.05).

**Figure 1:**
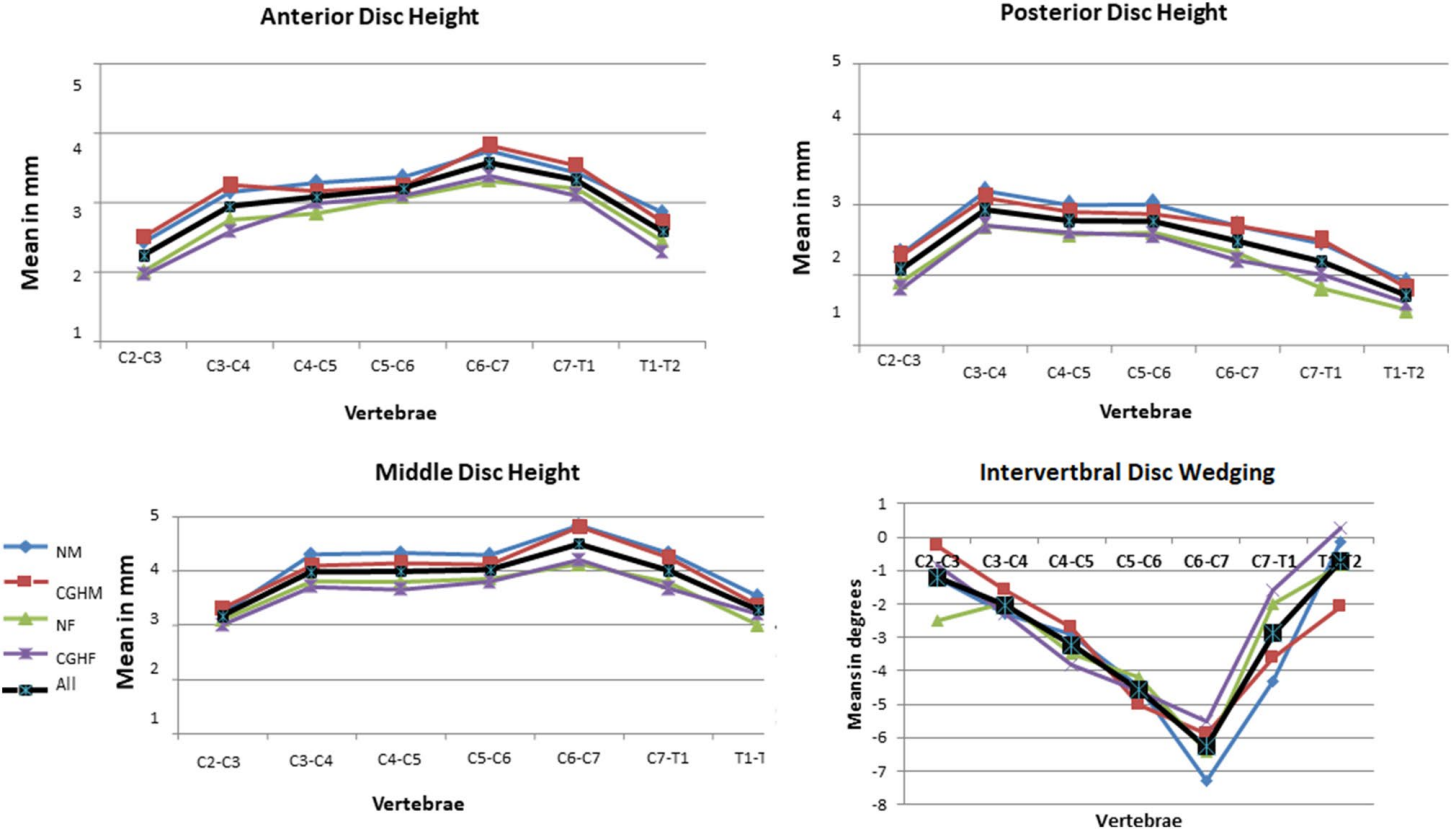
Intervertebral disc shape variation along the cervical spine. *NM* normal males, *CEHM* cervicogenic headaches males, *NF* Normal females, *CEHF* cervicogenic headaches females, *All* all individuals combined.

**Figure 2:**
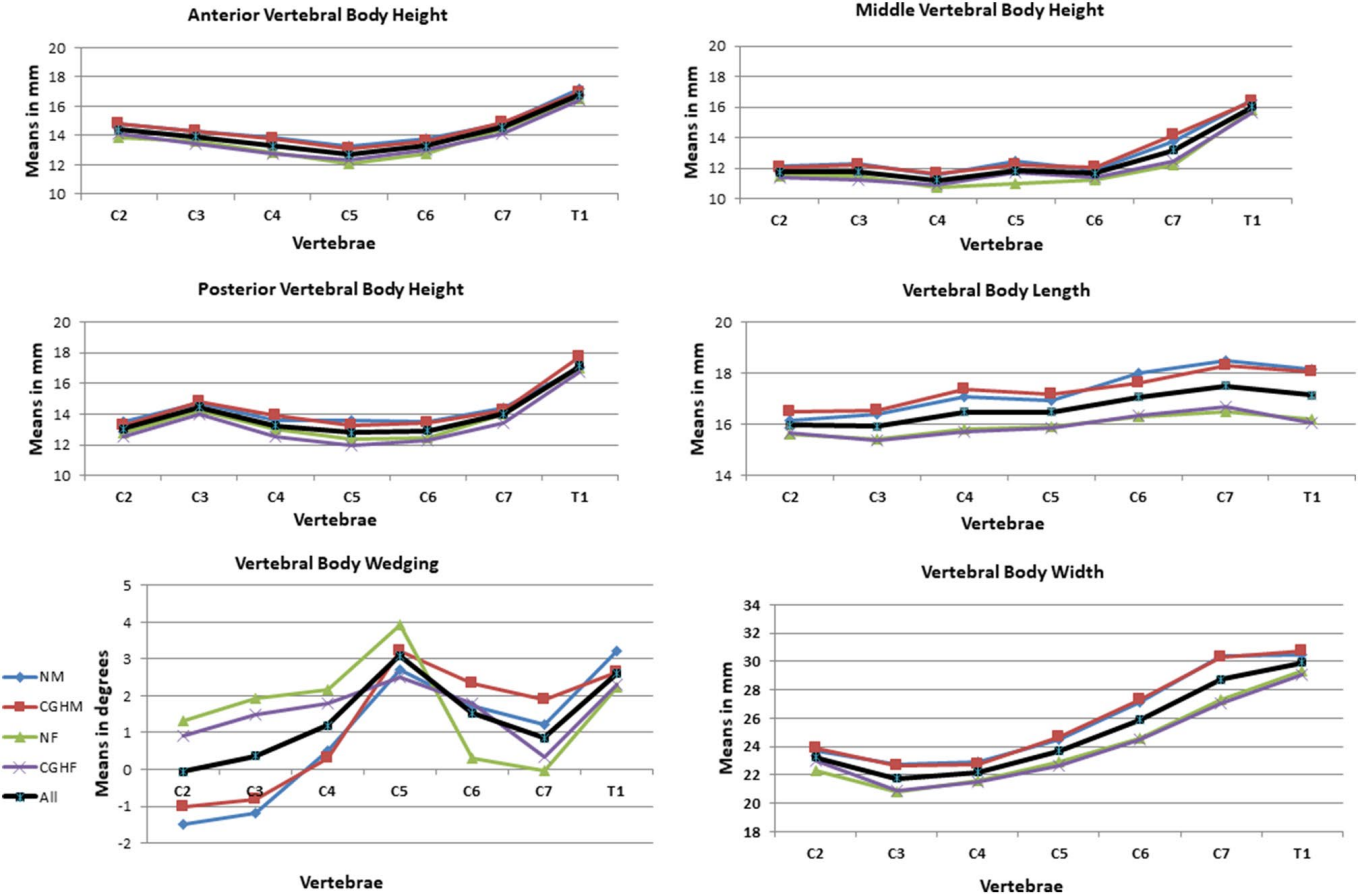
Vertebral body shape variation along the cervical spine. *NM* normal males, *CEHM* cervicogenic headaches males, *NF* Normal females, *CEHF* cervicogenic headaches females, *All* all individuals combined.

**Figure 3:**
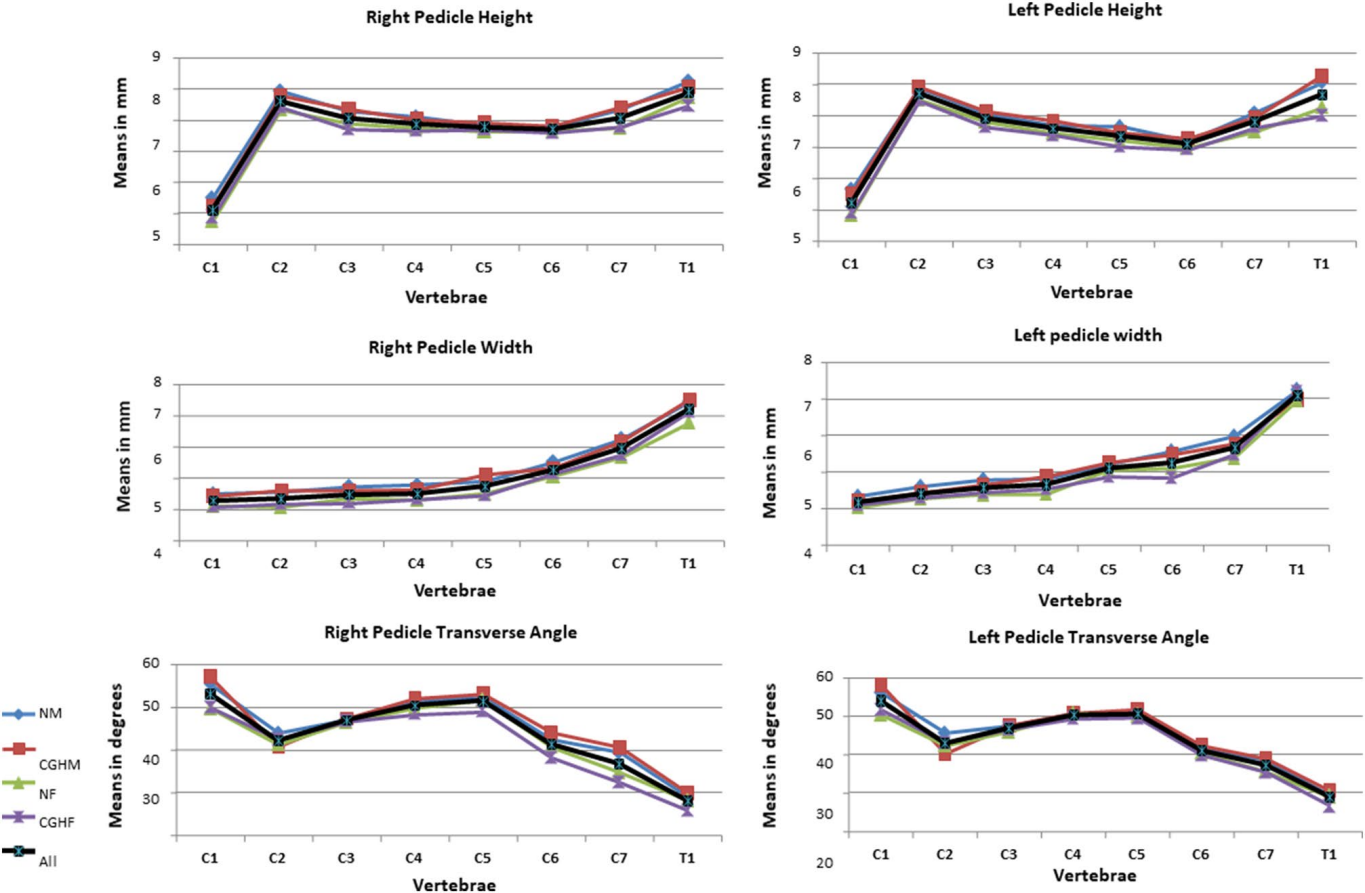
Pedicle shape variation along the cervical spine. *NM* normal males, *CEHM* cervicogenic headaches males, *NF* Normal females, *CEHF* cervicogenic headaches females, *All* all individuals combined.

**Figure 4:**
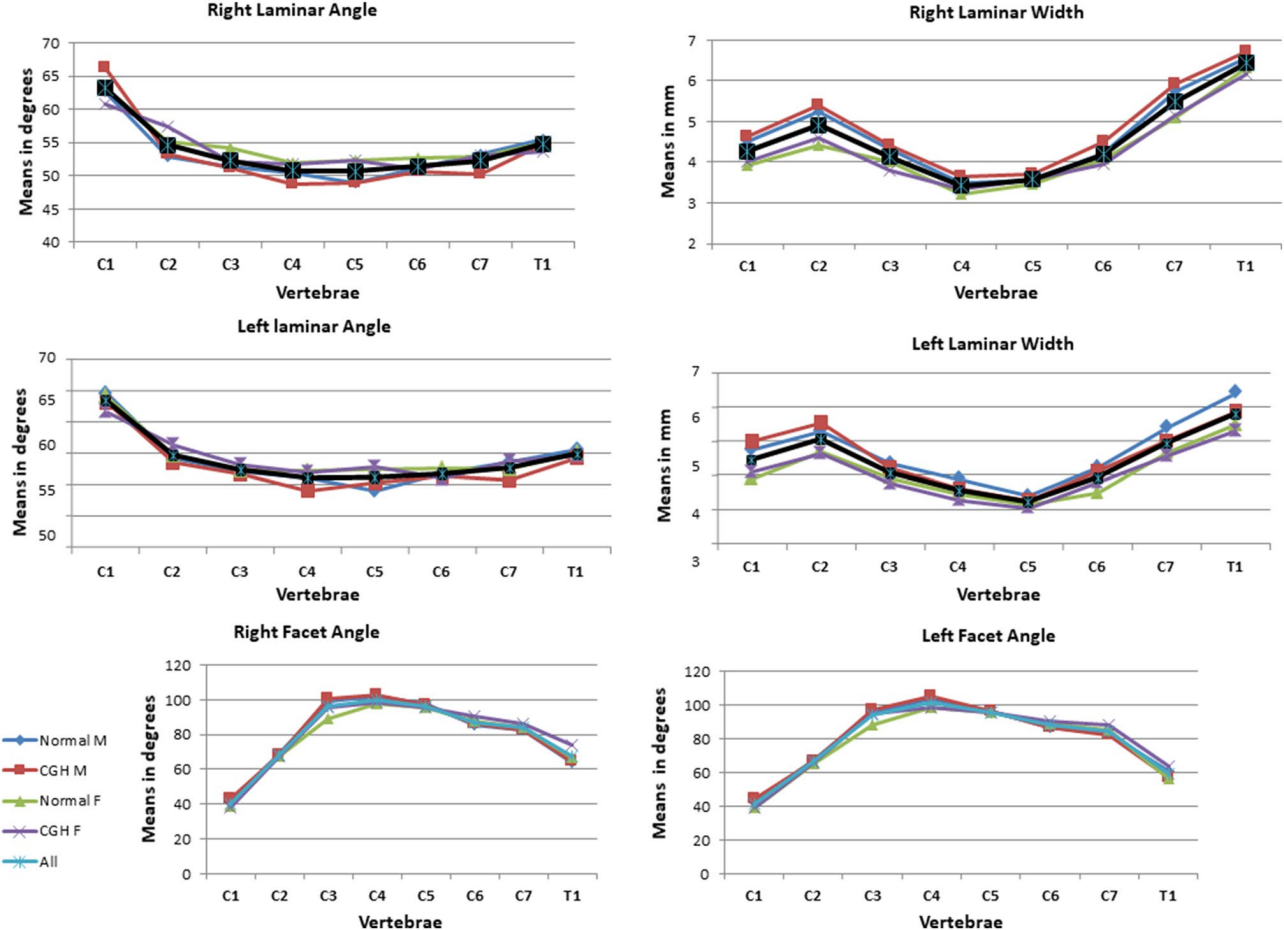
Lamina shape variation along the cervical spine*. NM* normal males, *CEHM* cervicogenic headaches males, *NF* Normal females, *CEHF* cervicogenic headaches females, *All* all individuals combined.

**Figure 5:**
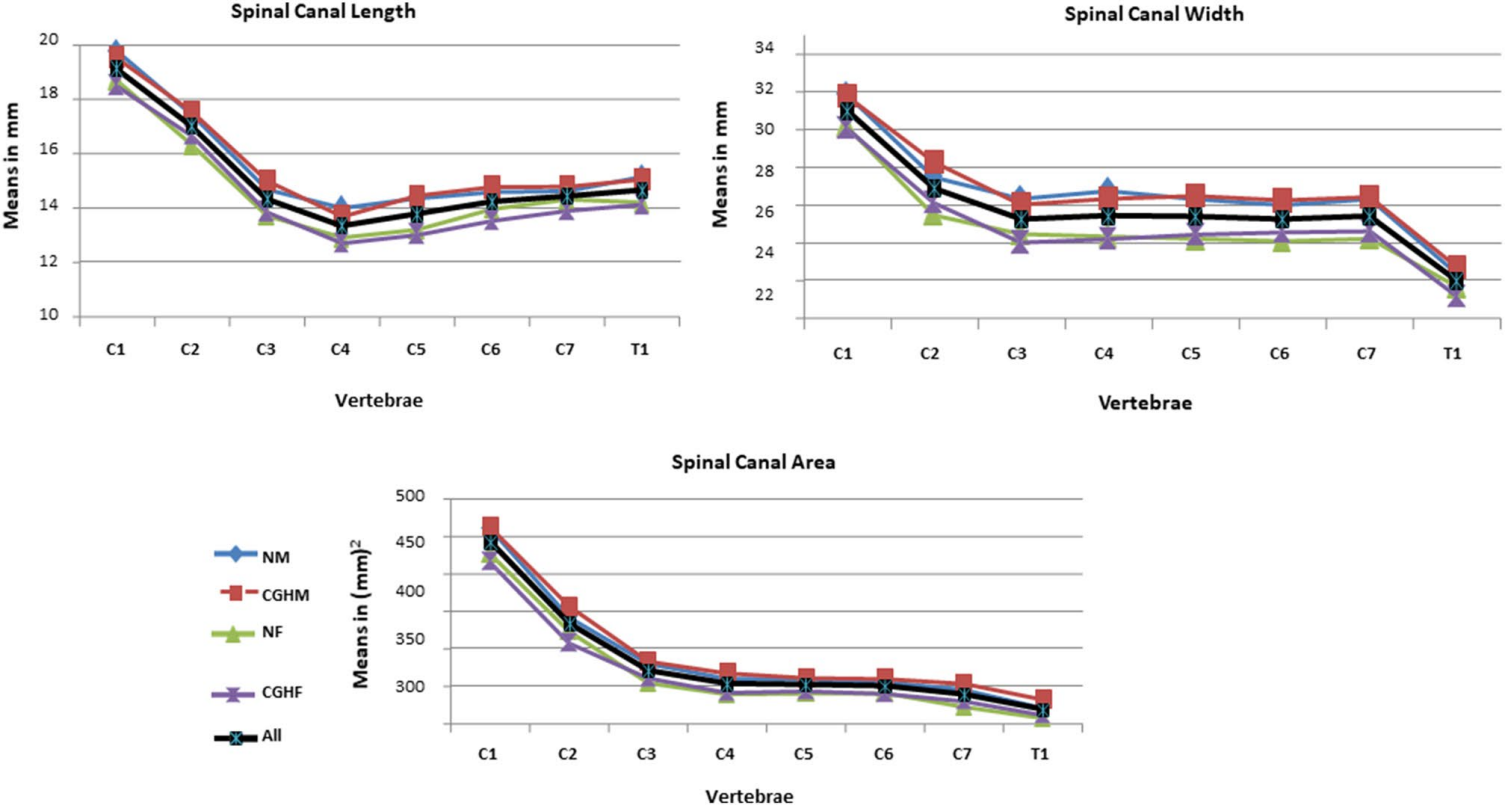
Spinal canal shape variation along the cervical spine. *NM* normal males, *CEHM* cervicogenic headaches males, *NF* Normal females, *CEHF* cervicogenic headaches females, *All* all individuals combined.

- No significant differences were found between the CEH and controls in any measured parameters (vertebral and IVD) concerning sex (t-test, p > 0.05) and age (ANOVA, p > 0.05) (Table 1).
- The shape variation of all measured parameters along the CEH and control groups’ cervical spine is presented in Figs. 1, 2, 3, 4, and 5. In general, a similar pattern of shape variation is shown in the two groups for all shape parameters as follows:
  - *Intervertebral disc (IVD)* In general, the anterior and middle IVD heights are approximately 2–4 mm, whereas the posterior heights are approximately 2–3 mm, producing a general lordotic IVD shape along the cervical spine. Their lowest values are usually at C2–3 and T1–2 (Fig. 1 and Table 1 in Appendix 2).
  - *Vertebral body (VB)* In general, the VB posterior and middle heights remain constant from C2 to C6 (approximately 12 mm); subsequently, they sharply increase by 2–4 mm towards T1 except for an increased posterior VB height at C3. The anterior VB heights first decrease from C2 to C5 (approximately 2 mm), followed by a continuous increase towards T1 (about 16 mm of height). In most cases, the cervical VBs are more kyphotic than lordotic in shape (approximately 2°) except for a clear lordotic VB at C2 (about 10° s). The VBs are always wider (between 22 and 30 mm) rather than longer (between 16 and 18 mm) along the cervical spine (Fig. 2 and Table 1 in Appendix 2).
  - *Pedicle* The pedicle is at its shorter, narrowest, and most frontally oriented values at C1 (approximately 4 mm for heights and widths and 50°–60° for orientation). Pedicle widths continuously increase from C2–T1 (from about 4 mm to 7 mm), whereas its heights decreased from C2–C6, followed by an increase to T1 (Fig. 3 and Tables 2 and 3 in Appendix 2).
  - *Lamina* The laminar widths increase from C1 (approximately 4 mm) to C2, gradually decreasing to C4-C5 (about 3–4 mm) and subsequently increase to T1 (about 6 mm). The laminar is most frontally oriented at C1 (approximately 60°–65°), then remaining almost constant from C2 to T1 (between 50° and 55°) (Fig. 4 and Table 2 in Appendix 2).
  - *Articular facet* the superior articular facets at C1 are in their most horizontal orientation (approximately 40° with the horizontal line), turning sagittally between C3 and C5 (approximately 100° with the horizontal line), followed by a more horizontal orientation towards T1 (approximately 67° with the horizontal line) (Fig. 4 and Table 4 in Appendix 2).
  - *Spinal canal* The spinal canal is always wider than longer along the cervical spine (approximately ∆10 mm between width and length at each level). Its lengths, widths, and area sharply decrease from C1 to C3–C4 and remain almost constant towards C7. Its widths decrease again at T1 (approximately 22 mm) (Fig. 5 and Table 5 in Appendix 2).
  - *Transverse foramen* The transverse foramen lengths and widths are at their highest values at C1 (approximately 6.5 mm), gradually decreasing along the cervical spine. In most vertebrae, the transverse foramen is wider than longer (approximately ∆1 mm) (Table 5 in Appendix 2).

**Table 1:** Subjects characteristics.

Variable (N = 80)	Gender	Group	Mean (SD)
Age	Males	Control	30.7 (6.9)
		CGH	30 (6.3)
	Females	Control	28 (6.7)
		CGH	29 (6.2)
Height	Males	Control	188 (7.5)
		CGH	189 (4.9)
	Females	Control	166.8 (4.9)*
		CGH	167 (4.9)*
Weight	Males	Control	83.8 (9.2)
		CGH	83.6 (6.3)
	Females	Control	60.8 (10.1)
		CGH	62.7 (10.8)
BMI	Males	Control	23.6 (1.9)
		CGH	23.4 (2.07)
	Females	Control	21.7(3.3)
		CGH	22.2 (3.7)

*SD* standard deviation, *CGH* cervicogenic headache, *BMI* Body mass index.

*p < 0.05.

## Discussion

This study assessed the associations between the shapes of all cervical vertebrae and their adjacent IVDs with CEH. We found that in subjects affected with CEH, these two structures are statistically indistinguishable from asymptomatic controls confirming their non-contribution as hypothesized. Also, their shape variation from C2 to C7 was identical between the two groups. We are unaware of other studies that have utilized such a thorough investigation into the spine’s morphology in subjects afflicted with CEH.

We purposefully aimed at a younger population to control for possible confounders of age-related degeneration in CEH. Our chosen subjects’ mean age was 30.7, and other researchers reported the mean onset of CEH to be 33^3^. This fact may limit the generalizability of our findings to subjects in their 30 s presenting with CEH, and it may also suggest different etiologies of CEH in older age groups. According to the present study, we conclude that the shape of the cervical vertebrae and their adjacent IVDs, do not seem to be major contributing factors in individuals in their late 20’s-mid 30’s affected with CEH. Therefore, it is reasonable to suggest that CTs should only be considered for excluding any possible systemic pathology in this population to avoid unnecessary exposure to ionizing radiation. Another strength of the study is its retrospective nature which allowed us to carefully select only the cases that adhered to the most demanding diagnostic criteria of CEH.

Noteworthy is that the spinal canal and transverse foramen were not statistically different between males and females in the CEH and control groups, although we observed a tendency for greater dimensions in males than in females at all levels. This tendency was significantly different in other studies for selected cervical vertebrae and specific ethnic groups (Europeans, Americans^23^, and Japanese^24^). This exceptional result could be related to the fact that we did not consider ethnicity as a covariant in the current study, and it is also possible that directly measuring dry vertebrae could end with different results^25^.

Since the shape variation of all measured anatomical parameters along the cervical spine was almost identical in the CEH and control group, it is reasonable to suggest that all these anatomical parameters’ functional aspects are similar. Although it is beyond this paper’s scope, one could logically conclude that having similar anatomical features that manifest similar shape variations along with a mobile unit such as the cervical region would indicate similar functions in the range of motion, velocity, and other kinematical or biomechanical parameters. One such parameter is that the IVDs are narrower at the upper cervical (C2–3) and upper thoracic (T1-T2) regions. This IVDs narrowing is probably related to the excessive mechanical stresses associated with the head-weight in the upper cervical area and the head and neck in the cervicothoracic junction^26^.

We acknowledge a few limitations to the current study. As we have previously noted, some researchers suggested that the etiology of CEH is related mainly to soft tissues, disc herniations, and other common age-related degenerative findings^2,17,27,28^. As we only included subjects who have undergone a CT scan, it may be possible that an MRI scan would have revealed soft tissue abnormalities in those subjects that CT scans cannot appropriately image. Accordingly, we advise the readers to interpret the bony morphology results from this study specifically with subjects in their late 20’s–mid 30’s. We suggest that future studies in this age group focus on MRI imaging, and older age groups’ studies incorporate both CT and MRI imaging when possible.

In conclusion, in subjects in their late 20’s–mid 30’s affected with CEH, the cervical spine’s vertebral and IVDs shapes and shape variations are not unique compared to asymptomatic controls. These results suggest that in this population, the bony and discal structures of the cervical spine can be excluded from the clinical investigation.

## Methods

### Human ethics

The study was approved by the Institutional Ethics Committee of “Clalit Health Services” (#CMC-0046-13) and by the institutional review board of Tel-Aviv University (Date: 24.2.2014) and was conducted according to the Good Clinical Practice guidelines (GCP). Due to the retrospective nature of the study, informed consent was waived by both committees.

### Study design

Observational, retrospective cross-sectional.

### Imaging equipment

The hospital where we obtained the CT scans uses a high-resolution CT scanner (Brilliance 64, Philips Medical Systems; voltage 120 kV, current 150–570 mA), enabling scan processing in all planes allowing optimal plane positioning for each 2D measurement.

### Study sample

We took a total of 19,040 measurements from 80 multi-planar (3D) reformations of plain CT images of 40 subjects affected with chronic CEH and 40 controls (20 males and 20 females in each group), aged 20–40 years. This age-range was specifically selected to control for the effect of degenerative changes. The 3D reformations were processed using the volume rendering method (Phillips Brilliance 64 CT, the thickness of sections: 1–2 mm, MAS: 80–250). All CT images for both groups were taken in the same position.

### Inclusion and exclusion criteria

The CTs were initially performed in the hospital to negate possible systemic pathologies due to chronic headaches (i.e., more than 3 months of continuing symptoms) rather than a diagnostic tool for CEH. The control group included individuals who had undergone a post-trauma (i.e., falls and road accidents) CT evaluation as a safety measure, yet had no objective pathological findings (Age-sex matched control group) nor suffered from any headaches as specifically reported and negated in their medical records. Inclusion criteria included subjects who were diagnosed with CEH by a physician after they received anesthetic blocks that abolished the pain entirely but transiently and scored positively on at least 4 points from the criteria described by Bogduk and Govind^21^: (a) unilateral head pain without side alternation, (b) pain triggered by neck movement or sustained postures, and reduced range of motion of 10 degrees in at least one cervical movement plane, (c) fluctuating pain episodes, (d) pain starting in the neck and spreading towards the occiput, (e) Various attack-related events: autonomic symptoms and signs, nausea, vomiting, ipsilateral edema and flushing in the peri-ocular area, dizziness, photophobia, phonophobia, or blurred vision in the ipsilateral eye^29^.

### Primary outcome measures

The medical files and the diagnoses were all granted between 2009–2015 and before the ICHD 3rd edition was published^5^. Exclusion criteria included any level of cervical intervertebral disc herniation, spondyloarthropathies, fractures or dislocations of the cervical spine, history of brain or spinal surgery, co-morbid neurologic diseases including cerebral infarction, neuropathy, or symptoms related to sensory or motor disorders (numbness, clumsiness, motor weakness, and gait disturbances).

**Figure 6:**
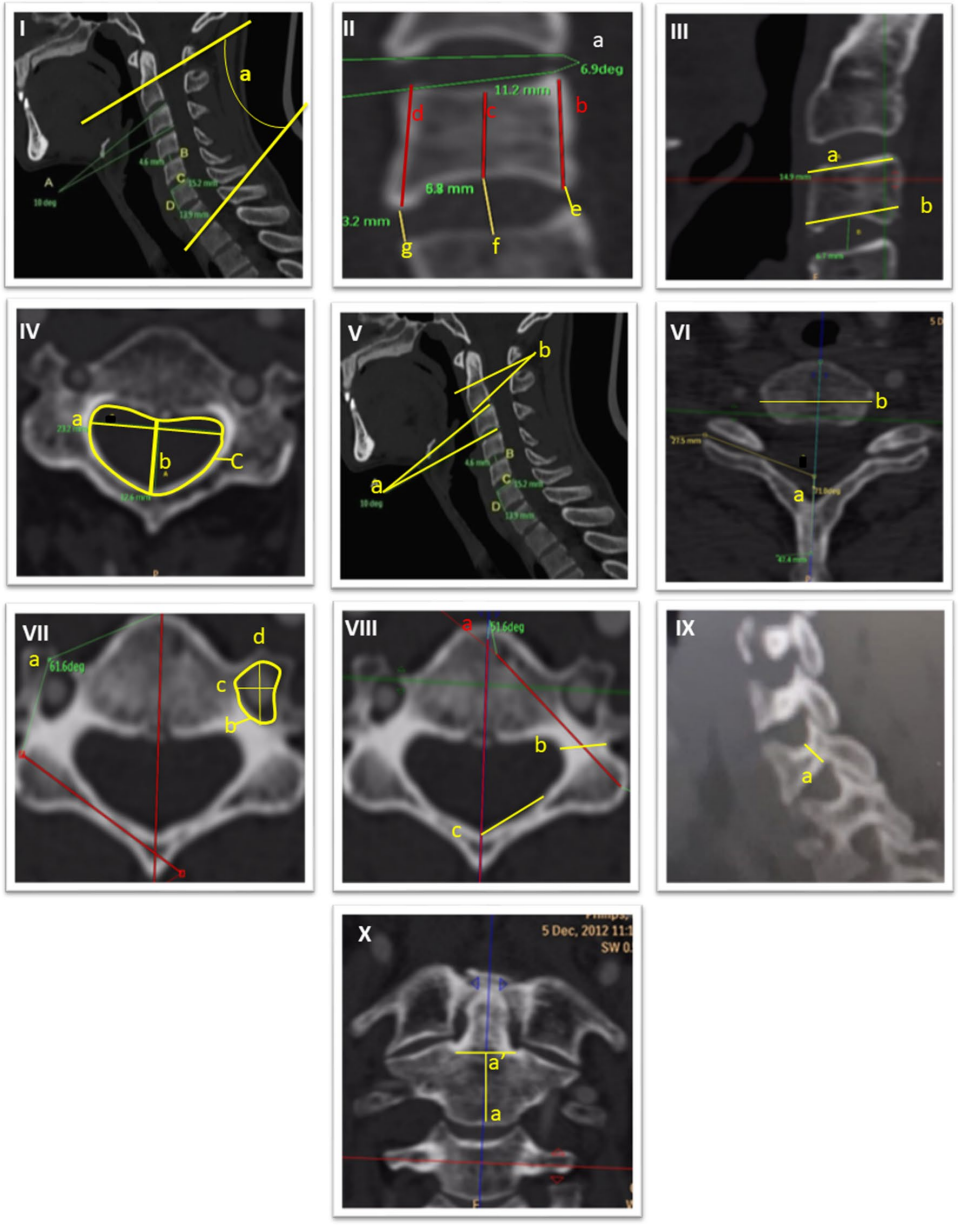
Morphometric measurements. (**I**) Supine cervical lordosis; (**II**) a—sagittal intervertebral disc wedging, b—posterior vertebral body (VB) height, c—middle VB height, d—anterior VB height, e—posterior disc height, f—middle disc height, g—anterior disc height; (**III**) a—superior VB length, b—inferior VB length; (**IV**) a–c—spinal canal width length and area, respectively; (**V**) sagittal VB wedging (a = kyphotic C3, b = lordotic C2); (**VI**) a—transverse facet angle, b—superior VB width; (**VII**) a—transverse laminar angle, b—transverse foramen area, c—transverse foramen width, d-transverse foramen length; (**VIII**) a—transverse pedicle angle, b—pedicle width, c—laminar length; (**IX**) a—pedicle height; (**X**) a–a’—anterior VB height of C2.

The following measurements were taken from the second cervical level to the first thoracic level (C2–T1), using a computerized program measuring angles and distances (Brilliance workspace portal, version V3.5.2.2254). We also included the first cervical vertebra when possible. All the following measurements are included in Fig. 6, and their complete definitions are included in Appendix 1:

- *Supine cervical lordosis* The angle drawn between the superior endplate line of C2 and inferior endplate line of C7.
- *Vertebral body (VB)* Anterior, middle, and posterior mid-sagittal heights, superior and inferior A-P extensions (i.e., lengths), superior mediolateral width, and sagittal wedging. For all cervical vertebrae, the uncinate processes were excluded from all measurements. For C2, the dens were excluded from all VB measurements.
- *Intervertebral disc* anterior middle and posterior heights and sagittal wedging.
- *Neural arch: Articular facet—*facet transverse angles; *Pedicles*—heights, widths, and transverse angles (for C1, the pedicle is considered as the posterior border of the lateral mass and for C1 as the most posterior border of the articular process); *Lamina*—mediolateral widths (for C1, the distance from the posterior tubercle to the lateral mass) and transverse angles; *Spinal canal*—lengths, widths and areas; *Transverse foramens*—lengths, widths, and areas.

### Reliability

We performed intra and inter-examiner reliability trials on 10 CT images. The intra-examiner reliability test was performed twice by the same examiner with an interval of one week in between. The inter-examiner reliability trials involved another tester who separately carried out the exact measurements using the same protocol and conditions.

### Data analysis

Data were analyzed using the Statistical Package for the Social Sciences, Version 23 (SPSS Inc., Chicago, IL, USA). Results of each test were assessed for mean, standard deviation (SD), and range. The Kolmogorov–Smirnov test evaluated the normal distribution of the variables. The paired t-tests determined any left–right differences between the means, averages, and SDs of the continuous data and to within the same subject. A one-way analysis of variances (ANOVA) determined the effect of independent variables on morphological parameters, and a post hoc Welch test was used to assess equal distribution and Bonferroni correction for multiple comparisons. P values < 0.05 were considered significant. The inter-intraclass correlations (ICC_3,1_) were calculated with 95% confidence intervals (CI) to establish Intra and inter-examiner reliability. A mean cutoff of ICC_3,1_ > 0.75, was chosen a priori and considered good^30–32^. P values < 0.05 were considered significant. We calculated the sample size using a desired minimal difference of 1 mm between the SDs of both groups and a two-tailed test. We based the desired difference upon similar previous published data^23^. For a power of 80% and an alpha of 0.05, the sample size recommended was 40 subjects in each group.

## Supplementary Information

Supplementary Information 1.

Supplementary Information 2.

## Data Availability

Data will be shared upon request. Please contact the corresponding author.

## References

[1] Fredriksen TA, Antonaci F, Sjaastad O (2015). Cervicogenic headache: Too important to be left un-diagnosed. J. Headache Pain.

[2] Olesen J (2013). The international classification of headache disorders, (beta version). Cephalalgia.

[3] Antonaci F, Sjaastad O (2011). Cervicogenic headache: A real headache. Curr. Neurol. Neurosci. Rep..

[4] Sjaastad O, Fredriksen T, Pfaffenrath V (1998). Cervicogenic headache: Diagnostic criteria. J. Headache Pain.

[5] Olesen J (2018). Headache classification committee of the international headache society (IHS) the international classification of headache disorders, asbtracts. Cephalalgia.

[6] Verma S, Tripathi M, Chandra PS (2021). Cervicogenic headache: Current perspectives. Neurol. India.

[7] Becker WJ (2010). Cervicogenic headache: Evidence that the neck is a pain generator. J. Headache Pain.

[8] Shen Y, Zhou Q, Li S, Jia Y, Qiu Z (2019). Clinical manifestations and imaging analysis of cervicogenic headache. Zhongguo Gu Shang.

[9] Fredriksen TA, Fougner R, Tangerud Å, Sjaastad O (1989). Cervicogenic headache. Radiological investigation concerning head/neck. Cephalalgia.

[10] Jansen J, Vadokas V, Vogelsang J (1998). Cervical peridural anaesthesia: An essential aid for the indication of surgical treatment of cervicogenic headache triggered by degenerative diseases of the cervical spine. Funct. Neurol..

[11] Shah P, Nafee A (1999). Clinical profile of headache and cranial neuralgias. J. Assoc. Physicians India.

[12] Sizer PS, Phelps V, Azevedo E, Haye A, Vaught M (2005). Diagnosis and management of cervicogenic headache. Pain Pract..

[13] Uthaikhup S, Assapun J, Kothan S, Watcharasaksilp K, Elliott JM (2017). Structural changes of the cervical muscles in elder women with cervicogenic headache. Musculoskelet. Sci. Pract..

[14] Fernandez-de-Las-Penas C (2007). Magnetic resonance imaging study of the morphometry of cervical extensor muscles in chronic tension-type headache. Cephalalgia.

[15] Chua NH, Suijlekom HV, Wilder-Smith OH, Vissers KC (2012). Understanding cervicogenic headache. Anesth Pain Med..

[16] Vincent MB (2011). Headache and neck. Curr. Pain Headache Rep..

[17] Garcia JD, Arnold S, Tetley K, Voight K, Frank RA (2016). Mobilization and manipulation of the cervical spine in patients with cervicogenic headache: Any scientific evidence?. Front. Neurol..

[18] Blumenfeld A, Siavoshi S (2018). The challenges of cervicogenic headache. Curr. Pain Headache Rep..

[19] Stemper BD, Pintar FA, Rao RD (2011). The influence of morphology on cervical injury characteristics. Spine.

[20] Mai JK, Paxinos G (2011). The Human Nervous System.

[21] Kerr FW (1972). Central relationships of trigeminal and cervical primary afferents in the spinal cord and medulla. Brain Res..

[22] Siddall PJ, Cousins MJ (1995). Pain mechanisms and management: An update. Clin. Exp. Pharmacol. Physiol..

[23] Ezra D (2019). The torg ratio of C3–C7 in African Americans and European Americans: A skeletal study. Clin. Anat..

[24] Ishikawa M, Matsumoto M, Fujimura Y, Chiba K, Toyama Y (2003). Changes of cervical spinal cord and cervical spinal canal with age in asymptomatic subjects. Spinal Cord.

[25] Masharawi Y (2008). Vertebral body shape variation in the thoracic and lumbar spine: Characterization of its asymmetry and wedging. Clin. Anat..

[26] Frobin W, Leivseth G, Biggemann M, Brinckmann P (2002). Vertebral height, disc height, posteroanterior displacement and dens–atlas gap in the cervical spine: Precision measurement protocol and normal data. Clin. Biomech..

[27] Rasmussen BK (1995). Epidemiology of headache. Cephalalgia.

[28] Fernández-de-las-Peñas C, Alonso-Blanco C, Cuadrado ML, Pareja JA (2005). Spinal manipulative therapy in the management of cervicogenic headache. J. Headache Pain.

[29] Bogduk N, Govind J (2009). Cervicogenic headache: an assessment of the evidence on clinical diagnosis, invasive tests, and treatment. Lancet Neurol..

[30] Shrout PE, Fleiss JL (1979). Intraclass correlations: Uses in assessing rater reliability. Psychol. Bull..

[31] Kramer MS, Feinstein AR (1981). Clinical biostatistics: LIV. The biostatistics of concordance. Clin. Pharmacol. Ther..

[32] Cicchetti DV (1994). Guidelines, criteria, and rules of thumb for evaluating normed and standardized assessment instruments in psychology. Psychol. Assess..

