# Supplementary material for: A comparative shape analysis of the cervical spine between individuals with cervicogenic headaches and asymptomatic controls
Source: Sci Rep. 2021 Sep 30;11:19413. doi: 10.1038/s41598-021-98981-y (PMC8484601; doi:10.1038/s41598-021-98981-y)
Supplement: Supplementary file 1 — Supplementary Information 1. [file 41598_2021_98981_MOESM1_ESM.docx]

| **Appendix 1. Measurements Definitions** | |
| --- | --- |
| **Vertebral Body:**  1. Anterior vertebral body height - the distance between the most anterior points of the superior and inferior end plates in the midsagittal plane.  2. Middle vertebral body height - the distance between the middle points of the superior and inferior endplates in the midsagittal plane.  3. Posterior vertebral body height -the distance between the most posterior points of the superior and inferior endplates in the midsagittal plane.  4. Superior vertebral body length - the distance between the most anterior point and the most posterior point of the superior vertebral body in the midsagittal plane.  5. Inferior vertebral body length -the distance between the most anterior point and the most posterior point of the inferior vertebral body in the midsagittal plane.  6. Sagittal vertebral body wedging - the angle between the superior and inferior endplates.  7. Superior vertebral body width - the distance between the most lateral vertebral body edges in the axial plane | **Neural Arch:**  *Pedicle Diameters:*  1. Pedicle height - the superior-inferior diameter of the pedicle isthmus at its narrowest point  2 .Pedicle width - the narrowest dimension of the pedicle isthmus in the transverse plane  3. Pedicle transverse angle - the angle between the pedicle length and the vertebral midline in the transverse plane.  *Lamina:*  1. Lamina width - the narrowest dimension of the Laminae in the transverse plane  2. Lamina angle - the angle between the laminae length and the vertebral midline in the transverse plane  *Facet:*  1. Transverse angle: the angle between the articular facet width and the sagittal plane.  *Spinal Canal Diameters:*  1. Spinal canal length - the distance between the anterior middle and posterior middle borders of the spinal canal in axial plane.  2. Spinal canal width - the distance between most lateral points of the spinal canal in axial plane.  3. Osseous spinal canal area- the area delineated by the bony structure of the spinal canal.  *Transverse Foramen:*  1. Transverse foramen length - The anterior-posterior distance in axial plane at mid-vertebral level.  2. Transverse foramen width - The medio-lateral distance in axial plane at mid-vertebral level.  3. Transverse foramen area - The area delineated by the bony structure of the spinal canal. |
| **Intervertebral Disc:**  1. Anterior intervertebral disc height - the distance between the anterior margins of the inferior endplate of the upper vertebral body and the superior endplate of the inferior vertebral body.  2. Middle intervertebral disc height - the longest distance between the superior endplate of the upper vertebral body and the inferior endplate of the lower vertebral body.  3. Posterior intervertebral disc height - the distance between the posterior margins of the inferior endplate of the upper vertebral body and the superior endplate of the inferior vertebral body.  4. Intervertebral disc sagittal wedging - the angle between the superior and inferior endplate of the intervertebral disc. |  |
| **Cervical Lordosis**  The angle between the line of the superior endplate of C2 and inferior endplate of C7. |  |
