# Supplementary material for: A comparative shape analysis of the cervical spine between individuals with cervicogenic headaches and asymptomatic controls
Source: Sci Rep. 2021 Sep 30;11:19413. doi: 10.1038/s41598-021-98981-y (PMC8484601; doi:10.1038/s41598-021-98981-y)
Supplement: Supplementary file 2 — Supplementary Information 2. [file 41598_2021_98981_MOESM2_ESM.docx]

| **Appendix 2 / Table 1. Vertebral body and Intervertebral disc measurements (CEH, n= 40; Control. n=40)** | | | | | | | | | | | | | |
| --- | --- | --- | --- | --- | --- | --- | --- | --- | --- | --- | --- | --- | --- |
| **Parameter** |  |  | **AVBH** | **MVBH** | **PVBH** | **SVBL** | **IVBL** | **VBW** | **VBwed** | **ADH** | | **MDH** | **PDH** |
| **Vertebra** | **Gender** | **Group** | **Mean**  **(mm)±SD** | **Mean**  **(mm)±SD** | **Mean**  **(mm)±SD** | **Mean**  **(mm)±SD** | **Mean**  **(mm)±SD** | **Mean**  **(mm)±SD** | **Mean**  **(degree)±SD** | **Mean**  **(mm)±SD** | | **Mean**  **(mm)±SD** | **Mean**  **(mm)±SD** |
| **C2** | **Males** | Cont. | 14.83±3.13 | 12.10±2.25 | 13.51±1.83 | 15.35±2.38 | 16.78±2.83 | 23.73±1.50 | -.48±3.03 | 2.56±0.62 | 3.21±0.79 | | 2.31±0.44 |
|  |  | CEH | 14.87±3.15 | 12.04±2.71 | 13.24±0.50 | 15.45±2.14 | 16.67±2.47 | 23.87±1.76 | -.25±3.93 | 2.65±0.51 | 3.32±0.70 | | 2.28±0.50 |
|  | **Females** | Cont. | 13.85±3.08 | 11.50±2.76 | 12.77±2.66 | 14.84±2.16 | 16.28±2.93 | 22.30±1.30 | -.29±3.94 | 2.10±0.73 | 3.09±1.00 | | 1.90±0.48 |
|  |  | CEH | 14.10±3.42 | 11.42±2.97 | 12.56±0.43 | 14.90±2.45 | 16.23±2.46 | 22.96±1.18 | -.58±4.88 | 2.05±0.80 | 3.00±0.86 | | 1.80±0.43 |
| **C3** | **Males** | Cont. | 14.32±1.76 | 12.32±1.23 | 14.75±1.61 | 16.47±2.87 | 17.13±2.17 | 22.70±1.71 | 1.85±3.98 | 3.35±1.06 | 4.30±0.86 | | 3.20±0.28 |
|  |  | CEH | 14.27±2.20 | 12.24±2.04 | 14.84±2.09 | 16.51±2.77 | 17.18±2.15 | 22.63±2.33 | 1.25±4.04 | 3.47±1.14 | 4.10±0.85 | | 3.10±0.37 |
|  | **Females** | Cont. | 13.62±1.62 | 11.45±1.44 | 14.22±1.39 | 15.77±2.20 | 16.73±2.90 | 20.80±2.22 | 2.54±3.41 | 2.92±0.96 | 3.80±1.09 | | 2.70±0.90 |
|  |  | CEH | 13.40±1.60 | 11.21±1.30 | 14.03±1.34 | 16.00±2.62 | 16.85±2.02 | 20.88±2.32 | 2.16±3.87 | 2.73±0.86 | 3.70±0.97 | | 2.70±0.25 |
| **C4** | **Males** | Cont. | 13.82±1.19 | 11.55±0.88 | 13.55±1.16 | 16.98±2.98 | 17.42±2.14 | 22.90±1.61 | 1.31±3.16 | 3.50±1.20 | 4.33±1.05 | | 3.00±1.19 |
|  |  | CEH | 13.75±1.16 | 11.67±0.87 | 13.88±1.05 | 16.83±2.95 | 17.50±2.27 | 22.69±1.64 | 1.73±3.36 | 3.37±1.37 | 4.15±1.26 | | 2.90±1.23 |
|  | **Females** | Cont. | 12.80±1.09 | 10.78±0.96 | 12.98±1.12 | 16.40±3.41 | 17.10±2.46 | 21.60±2.04 | 0.66±3.74 | 3.02±1.60 | 3.79±1.20 | | 2.57±1.42 |
|  |  | CEH | 12.73±1.24 | 10.92±1.50 | 12.51±1.19 | 16.60±2.87 | 17.06±2.03 | 21.53±1.81 | 0.70±2.00 | 3.18±1.08 | 3.65±0.82 | | 2.60±0.93 |
| **C5** | **Males** | Cont. | 13.23±0.98 | 12.45±1.02 | 13.62±1.23 | 17.05±1.97 | 17.82±1.64 | 24.50±2.65 | 1.98±3.72 | 3.59±0.99 | 4.29±0.76 | | 3.01±0.54 |
|  |  | CEH | 13.10±1.01 | 12.22±1.00 | 13.24±1.14 | 17.00±2.12 | 17.69±1.61 | 24.63±2.60 | 2.40±3.68 | 3.45±1.07 | 4.12±0.90 | | 2.87±0.62 |
|  | **Females** | Cont. | 12.10±1.06 | 11.00±1.23 | 12.35±1.22 | 16.61±2.54 | 17.15±1.97 | 22.90±2.80 | 2.17±2.24 | 3.27±1.34 | 3.85±1.02 | | 2.60±1.06 |
|  |  | CEH | 12.31±0.99 | 11.72±1.26 | 12.00±1.28 | 16.53±2.08 | 17.30±1.69 | 22.67±2.65 | 1.80±2.17 | 3.30±1.02 | 3.80±0.71 | | 2.56±0.61 |
| **C6** | **Males** | Cont. | 13.77±0.78 | 11.92±0.95 | 13.50±1.83 | 17.06±2.09 | 18.03±2.13 | 27.10±2.10 | 1.20±2.50 | 4.00±0.57 | 4.84±0.51 | | 2.70±0.59 |
|  |  | CEH | 13.64±1.09 | 12.03±1.18 | 13.43±3.69 | 17.13±2.08 | 18.11±2.12 | 27.30±2.20 | 1.76±2.76 | 4.10±0.76 | 3.21±0.79 | | 2.70±0.75 |
|  | **Females** | Cont. | 12.77±1.00 | 11.21±1.07 | 12.48±3.14 | 16.52±2.77 | 17.68±2.14 | 24.60±2.06 | 1.64±3.09 | 3.53±0.70 | 3.32±0.70 | | 2.30±0.65 |
|  |  | CEH | 12.98±0.99 | 11.44±1.03 | 12.30±3.17 | 16.66±2.17 | 17.76±1.75 | 24.52±2.75 | 2.01±2.01 | 3.61±0.62 | 3.09±1.00 | | 2.20±0.63 |
| **C7** | **Males** | Cont. | 14.81±0.87 | 13.80±0.73 | 14.42±1.05 | 17.21±1.77 | 18.32±3.38 | 30.40±2.81 | -.47±2.32 | 3.66±0.73 | 3.00±0.86 | | 2.45±0.56 |
|  |  | CEH | 14.89±0.98 | 14.2±1.040 | 14.23±1.03 | 17.32±1.74 | 18.45±3.09 | 30.26±2.76 | -.39±2.30 | 3.78± 0.64 | 4.30±0.86 | | 2.50±0.53 |
|  | **Females** | Cont. | 14.42±0.93 | 12.20±0.80 | 14.00±1.15 | 16.72±1.92 | 17.99±2.39 | 27.33±2.55 | -.57±1.98 | 3.42±0.76 | 4.10±0.85 | | 1.80±0.67 |
|  |  | CEH | 14.12±0.96 | 12.50±0.93 | 13.45±1.24 | 16.70±1.57 | 17.79±2.07 | 27.00±2.05 | -.04±2.09 | 3.30±0.67 | 3.80±1.09 | | 2.00±0.55 |
| **T1**  CEH = cervicogenic headache; Cont. = control; AVBH –anterior vertebral body height; MVBH = middle vertebral body height; PVBH = posterior cervical vertebral body height; SVBL = superior vertebral body length; IVBL = inferior vertebral body length; VBW = vertebral body width; VBwed. = vertebral body sagittal wedging (negative value = lordotic wedging, positive value = kyphotic wedging); ADH = anterior disc height; MDH = middle disc height; PDH = posterior disc height C2-C7 = cervical vertebrae, T1 = thoracic vertebra; mm = millimeters | **Males** | Cont. | 17.17±1.06 | 16.44±1.33 | 16.99±1.37 | 17.10±2.05 | 18.22±4.74 | 30.50±2.04 | 4.17±6.03 | 3.03±1.02 | 3.70±0.97 | | 1.90±0.55 |
|  |  | CEH | 16.92±1.17 | 16.36±1.44 | 17.75±1.40 | 17.28±1.78 | 18.19±4.66 | 30.76±2.61 | 4.88±5.38 | 2.88±1.16 | 4.33±1.05 | | 1.82± 0.63 |
|  | **Females** | Cont. | 16.52±1.21 | 15.79±1.22 | 16.97±1.14 | 16.88±2.03 | 18.00±2.47 | 29.31±2.16 | 2.66±2.91 | 2.58±0.91 | 4.15±1.26 | | 1.50±0.71 |
|  |  | CEH | 16.45±1.19 | 15.61±1.39 | 16.78±1.11 | 16.80±1.83 | 17.85±2.22 | 29.09±2.14 | 1.97±3.32 | 2.40±0.80 | 3.79±1.20 | | 1.60± 0.49 |

| **Appendix 2 / Table 2: Pedicle and laminar linear measurments (CEH, n= 40; Control. n=40)** | | | | | | | |
| --- | --- | --- | --- | --- | --- | --- | --- |
| **Parameter** |  |  | **RPH** | **LPH** | **RPW** | **LPW** |  |
| **Vertebra** | **Gender** | **Group** | **Mean**  **(mm)±SD** | **Mean**  **(mm)±SD** | **Mean**  **(mm)±SD** | **Mean**  **(mm)±SD** |  |
| **C1** | **Males** | Cont. | 4.50±1.19 | 4.67±1.66 | 4.5±0.78 | 4.33±1.00 |  |
|  |  | CEH | 4.23±0.75 | 4.52±2.66 | 4.43±0.81 | 4.21±0.96 |  |
|  | **Females** | Cont. | 3.75±1.74 | 3.84±1.84 | 4.1±0.76 | 4.04±0.70 |  |
|  |  | CEH | 3.87±2.02 | 3.90±2.56 | 4.09±0.76 | 4.11±0.85 |  |
| **C2** | **Males** | Cont. | 7.92±1.15 | 7.86±1.31 | 4.56±0.65 | 4.60±0.47 |  |
|  |  | CEH | 7.78±2.23 | 7.91±1.22 | 4.61±0.51 | 4.43±0.64 |  |
|  | **Females** | Cont. | 7.33±0.99 | 7.52±1.28 | 4.05±0.52 | 4.28±0.85 |  |
|  |  | CEH | 7.42±1.79 | 7.49±1.37 | 4.19±0.46 | 4.30±0.64 |  |
| **C3** | **Males** | Cont. | 7.27±2.50 | 7.05±0.89 | 4.72±0.50 | 4.78±0.91 |  |
|  |  | CEH | 7.35±1.93 | 7.13±0.76 | 4.61±0.49 | 4.65±0.62 |  |
|  | **Females** | Cont. | 6.89±2.09 | 6.81±0.86 | 4.34±0.70 | 4.39±0.49 |  |
|  |  | CEH | 6.71±1.38 | 6.64±0.9 | 4.21±0.56 | 4.44±0.65 |  |
| **C4** | **Males** | Cont. | 7.10±2.33 | 6.69±0.44 | 4.79±0.47 | 4.81±1.04 |  |
|  |  | CEH | 7.01±2.81 | 6.84±0.48 | 4.63±0.56 | 4.88±0.72 |  |
|  | **Females** | Cont. | 6.74±2.13 | 6.45±0.47 | 4.29±0.75 | 4.40±0.72 |  |
|  |  | CEH | 6.66±1.99 | 6.39±0.55 | 4.33±0.58 | 4.54±0.78 |  |
| **C5** | **Males** | Cont. | 6.82±1.83 | 6.66±0.53 | 4.90±1.10 | 5.21±1.18 |  |
|  |  | CEH | 6.91±2.25 | 6.46±0.60 | 5.11±0.76 | 5.26±0.81 |  |
|  | **Females** | Cont. | 6.63±2.88 | 6.22±0.5 | 4.50±0.58 | 5.06±0.61 |  |
|  |  | CEH | 6.70±1.24 | 6.01±0.54 | 4.46±0.87 | 4.87±0.92 |  |
| **C6** | **Males** | Cont. | 6.77±2.12 | 6.19±0.72 | 5.51±1.21 | 5.56±0.97 |  |
|  |  | CEH | 6.80±2.56 | 6.28±0.73 | 5.32±0.90 | 5.47±0.80 |  |
|  | **Females** | Cont. | 6.64±2.38 | 5.98±0.67 | 5.05±0.72 | 5.11±0.69 |  |
|  |  | CEH | 6.60±2.73 | 5.92±0.64 | 5.14±0.90 | 4.84±0.70 |  |
| **C7** | **Males** | Cont. | 7.33±1.57 | 7.10±1.07 | 6.24±1.02 | 5.98±1.10 |  |
|  |  | CEH | 7.41±1.88 | 6.95±1.07 | 6.17±1.03 | 5.77±1.04 |  |
|  | **Females** | Cont. | 6.75±2.00 | 6.50±1.01 | 5.65±1.06 | 5.40±0.82 |  |
|  |  | CEH | 6.78±1.93 | 6.63±0.92 | 5.73±0.89 | 5.48±0.86 |  |
| **T1** | **Males** | Cont. | 8.23±1.26 | 8.04±2.36 | 7.44±0.94 | 7.23±1.03 |  |
|  |  | CEH | 8.05±0.99 | 8.28±2.35 | 7.50±0.90 | 6.98±0.90 |  |
|  | **Females** | Cont. | 7.73±1.80 | 7.27±2.06 | 6.76±1.06 | 6.96±0.94 |  |
|  |  | CEH | 7.46±1.57 | 7.01±1.87 | 7.10±0.80 | 7.18±0.90 |  |
| CEH = cervicogenic headache; Cont. = control; RPH = right pedicle height; LPH = left pedicle height; RPW = right pedicle width; LPW = left pedicle width; RLW = right laminar width; mm = milimeters ; SD = standard deviation; C1-C7 = cervical vertebrae, T1 = thoracic vertebra | | | | | | |  |

| **Appendix 2 / Table 3. Pedicle angular measurements (Means in degrees) ± Standard deviation (CEH, n= 40; Control. n=40)** | | | | | | | |
| --- | --- | --- | --- | --- | --- | --- | --- |
|  |  |  | **RPTA** | **LPTA** | **RLTA** | **LLTA** |  |
| **C1** | **Males** | Cont. | 55.37±5.81 | 56.05±5.79 | 62.64±4.24 | 64.50±4.60 |  |
|  |  | CEH | 57.29±4.80 | 58.15±4.80 | 66.20±5.02 | 63.10±5.22 |  |
|  | **Females** | Cont. | 49.70±6.03 | 50.30±5.98 | 63.15±3.76 | 64.09±3.14 |  |
|  |  | CEH | 50.10±6.60 | 51.68±6.30 | 60.70±4.36 | 61.60± 4.34 |  |
| **C2** | **Males** | Cont. | 43.90±4.81 | 45.50±4.69 | 52.90±2.82 | 54.10±1.87 |  |
|  |  | CEH | 40.80±5.20 | 40.34±5.20 | 53.22±2.14 | 53.50±3.67 |  |
|  | **Females** | Cont. | 41.20±4.19 | 42.30±4.51 | 55.10±3.39 | 54.60±4.04 |  |
|  |  | CEH | 42.50±3.70 | 43.00±4.80 | 57.30±4.67 | 56.22±4.9 4 |  |
| **C3** | **Males** | Cont. | 47.01±2.98 | 47.37±2.68 | 51.45±3.07 | 52.00±2.58 |  |
|  |  | CEH | 47.43±3.02 | 47.71±2.82 | 51.12±4.46 | 51.66±3.95 |  |
|  | **Females** | Cont. | 46.48±3.87 | 45.96±3.31 | 54.14±3.38 | 52.22±2.54 |  |
|  |  | CEH | 46.74±2.91 | 46.61±2.56 | 52.16±4.26 | .0153±3.33 |  |
| **C4** | **Males** | Cont. | 51.47±4.08 | 50.62±4.64 | 50.50±4.23 | 50.99±4.61 |  |
|  |  | CEH | 52.19±3.38 | 51.84±4.30 | 48.68±4.62 | 49.00±3.92 |  |
|  | **Females** | Cont. | 49.79±4.80 | 50.63±4.37 | 51.97±4.16 | 52.10±3.76 |  |
|  |  | CEH | 48.40±6.32 | 50.31±3.30 | 51.68±3.92 | 51.78±4.22 |  |
| **C5** | **Males** | Cont. | 52.27±4.25 | 50.69±4.71 | 48.90±4.03 | 48.90±5.27 |  |
|  |  | CEH | 53.22±4.70 | 51.86±4.70 | 49.00±2.91 | 50.30±4.24 |  |
|  | **Females** | Cont. | 51.64±5.28 | 50.22±4.31 | 52.32±4.10 | 52.32±5.03 |  |
|  |  | CEH | 49.02±3.40 | 49.60±3.40 | 52.34±3.50 | 52.70±3.69 |  |
| **C6** | **Males** | Cont. | 42.34±9.65 | 41.25±6.29 | 51.20±4.33 | 51.76±3.41 |  |
|  |  | CEH | 44.14±8.80 | 42.38±6.40 | 50.72±3.75 | 51.34±3.81 |  |
|  | **Females** | Cont. | 40.55±9.17 | 40.31±6.30 | 52.70±3.96 | 52.50±4.38 |  |
|  |  | CEH | 38.13±9.40 | 39.79±6.80 | 50.97±4.88 | 50.88±3.44 |  |
| **C7** | **Males** | Cont. | 39.37±9.95 | 38.46±6.44 | 53.20±4.29 | 53.50±4.91 |  |
|  |  | CEH | 40.67±9.00 | 39.01±6.10 | 50.24±3.68 | 50.70±3.26 |  |
|  | **Females** | Cont. | 34.59±9.53 | 35.52±4.45 | 52.80±4.31 | 52.40±3.89 |  |
|  |  | CEH | 32.29±9.20 | 35.24±4.00 | 53.00±4.51 | 53.67±4.32 |  |
| **T1** | **Males** | Cont. | 28.83±7.52 | 29.01±7.05 | 55.54±3.38 | 55.50± 2.66 |  |
|  |  | CEH | 29.77±7.70 | 30.56±6.60 | 54.81±4.15 | 54.22±3.75 |  |
|  | **Females** | Cont. | 29.66±8.37 | 28.90±8.17 | 55.02±3.94 | 55.00±2.94 |  |
|  |  | CEH | 25.60±7.40 | 26.44±7.50 | 53.70±4.22 | 54.52±3.28 |  |
| CEH = cervicogenic headache; Cont. = control; RPTA = right pedicle transverse angle; LPTS = left pedicle transverse angle; RLTA = right laminar transverse angle; LLTA = left laminar transverse angle; C1-C7 = cervical vertebrae, T1 = thoracic vertebra | | | | | | | |

| **Appendix 2/ Table 4. Articular facet angular measurements (CEH, n= 40; Control. n=40)** | | | | |
| --- | --- | --- | --- | --- |
| **Parameter** |  |  | **LFA** | **RFA** |
| **Vertebra** | **Gender** | **Group** | **Mean**  **(degrees)±SD** | **Mean**  **(degrees)±SD** |
| **C1** | **Males** | Cont. | 42.0±4.3 | 41.2±3.9 |
|  |  | CEH | 44.2±3.9 | 43.1±4.1 |
| **C1** | **Females** | Cont. | 38.9±3.1 | 38.3±3.9 |
|  |  | CEH | 39.0±3.2 | 38.3±3.9 |
|  | **Males** | Cont. | 67.3±5.6 | 68.8±6.7 |
| **C2** |  | CEH | 66.4±5.4 | 67.9±6.6 |
|  | **Females** | Cont. | 65.2±5.3 | 67.2±6.4 |
|  |  | CEH | 66.0±6.5 | 67.7±6.9 |
|  | **Males** | Cont. | 95.7±7.8 | 99.5±9.9 |
| **C3** |  | CEH | 96.7±7.4 | 100.2±11.1 |
|  | **Females** | Cont. | 88.0±8.9 | 89.2±9.1 |
|  |  | CEH | 94.3±7.9 | 95.8±8.7 |
|  | **Males** | Cont. | 103.4±10.4 | 102±9.9 |
| **C4** |  | CEH | 104.8±9/9 | 102±9.1 |
|  | **Females** | Cont. | 98.3±9.8 | 97.5±8.9 |
|  |  | CEH | 98.2±8.9 | 98.4±9.9 |
|  | **Males** | Cont. | 96.2±9.8 | 97.5±9.5 |
| **C5** |  | CEH | 96.0±9.0 | 97.3±8.9 |
|  | **Females** | Cont. | 95.3±8.9 | 95.4±9.8 |
|  |  | CEH | 95.6±9.2 | 95.4±9.3 |
|  | **Males** | Cont. | 87.0±8/6 | 85.0±8.7 |
| **C6** |  | CEH | 86.6±8.4 | 86.1±8.8 |
|  | **Females** | Cont. | 89.0±7.8 | 87.7±8.9 |
|  |  | CEH | 90.0±8.7 | 90.2±7.9 |
|  | **Males** | Cont. | 82.8±8.4 | 81.5±8.5 |
| **C7** |  | CEH | 82.4±8.7 | 83.0±7.9 |
|  | **Females** | Cont. | 42.0±5.0 | 41.2±3.9 |
|  |  | CEH | 44.2±3.9 | 43.1±5.0 |
| **T1** | **Males** | Cont. | 38.9±4.9 | 38.3±3.9 |
| **T1** |  | CEH | 39.0±3.7 | 38.3±4.0 |
|  | **Females** | Cont. | 67.3±6.4 | 68.8±5.9 |
|  |  | CEH | 66.4±6.5 | 67.9±5.7 |
| Cont. = controls; CEH = cervicogenic headache; LFA = left facet angle; RFA = right facet angle; C1-C7 = cervical vertebrae, T1 = thoracic vertebra | | | | |

| **Appendix 2 / Table 5. Spinal canal and transverse foramen measurements (CEH, n= 40; Control. n=40)** | | | | | | | | | | | | | |
| --- | --- | --- | --- | --- | --- | --- | --- | --- | --- | --- | --- | --- | --- |
| **Parameter** |  |  | **SCL** | **SCW** | **SCA** | | **RTFL** | **LTFL** | **RTFW** | **LTFW** | **RTFA** | **LTFA** |  |
| **Vertebra** | **Gender** | **Group** | **Mean**  **(mm)±SD** | **Mean**  **(mm)±SD** | **Mean**  **(mm^2^)±SD** | | **Mean**  **(mm)±SD** | **Mean**  **(mm)±SD** | **Mean**  **(mm)±SD** | **Mean**  **(mm)±SD** | **Mean**  **(mm^2^)±SD** | **Mean**  **(mm^2^)±SD** |  |
| **C1** | **Males** | Cont. | 19.76±0.84 | 31.90±2.55 | | 460.5±36.27 | 6.87±1.89 | 6.78±0.14 | 6.74±0.79 | 6.62±1.75 | 33.36±9.27 | 34.82±8.22 |  |
|  |  | CEH | 19.53±1.39 | 31.79±1.32 | | 462.5±30.91 | 6.86±0.96 | 6.65±1.37 | 6.72±0.38 | 6.57±1.66 | 32.89±11.28 | 34.49±7.12 |  |
|  | **Females** | Cont. | 18.70±0.90 | 30.20±1.67 | | 426.2±29.90 | 6.37±1.82 | 6.23±1.75 | 6.49±0.44 | 6.30±0.89 | 30.30±8.01 | 31.44±9.40 |  |
|  |  | CEH | 18.50±1.06 | 30.09±1.09 | | 415.9±28.59 | 6.16±1.53 | 6.32±1.27 | 6.41±1.28 | 6.10±1.09 | 31.20±7.15 | 30.81±6.33 |  |
| **C2** | **Males** | Cont. | 17.42±1.57 | 27.50±2.17 | | 342.0±35.18 | 5.75±0.46 | 5.34±1.91 | 6.37±1.68 | 6.25±0.34 | 25.70±7.90 | 27.77±7.47 |  |
|  |  | CEH | 17.55±0.97 | 28.30±1.22 | | 356.8±20.52 | 5.60±1.31 | 5.60±0.53 | 6.22±1.22 | 6.21±1.78 | 26.05±6.46 | 27.37±6.34 |  |
|  | **Females** | Cont. | 16.34±1.21 | 25.50±1.93 | | 322.9±37.63 | 5.05±0.93 | 4.90±1.91 | 5.95±1.50 | 5.94±0.91 | 24.50±9.47 | 26.07±4.57 |  |
|  |  | CEH | 16.66±1.01 | 26.12±1.19 | | 307.1±27.26 | 5.25±1.89 | 4.60±1.59 | 5.74±1.20 | 5.94±0.62 | 24.10±9.35 | 25.76±5.86 |  |
| **C3** | **Males** | Cont. | 14.67±1.40 | 26.35±2.32 | | 280.1±35.36 | 5.01±0.64 | 5.01±0.71 | 6.08±0.94 | 6.20±1.67 | 21.21±6.33 | 22.96±6.94 |  |
|  |  | CEH | 14.99±1.12 | 26.04±1.41 | | 282.8±21.56 | 5.20±1.36 | 4.60±1.14 | 6.08±0.93 | 6.19±1.49 | 21.78±6.97 | 23.14±4.96 |  |
|  | **Females** | Cont. | 13.72±1.29 | 24.50±2.36 | | 254.0±31.00 | 4.36±0.74 | 4.36±1.67 | 5.91±1.91 | 5.87±1.75 | 20.04±7.35 | 21.84±7.95 |  |
|  |  | CEH | 13.84±1.51 | 24.01±1.24 | | 260.0±22.52 | 4.51±1.24 | 4.02±0.49 | 5.60±1.55 | 5.62±1.27 | 20.12±7.47 | 21.53±9.23 |  |
| **C4** | **Males** | Cont. | 13.99±1.83 | 26.77±2.66 | | 260.1±23.77 | 4.77±1.46 | 4.77±1.39 | 5.88±1.81 | 6.03±0.53 | 21.28±5.13 | 22.25±8.12 |  |
|  |  | CEH | 13.67±1.27 | 26.35±1.55 | | 267.4±20.86 | 4.89±0.83 | 4.60±1.74 | 5.94±0.59 | 5.90±1.91 | 21.47±4.59 | 22.32±6.28 |  |
|  | **Females** | Cont. | 12.92±1.56 | 24.36±1.20 | | 239.2±25.23 | 4.20±0.36 | 4.20±0.78 | 5.69±1.72 | 5.76±1.59 | 20.00±7.64 | 20.70±8.23 |  |
|  |  | CEH | 12.69±1.47 | 24.20±1.01 | | 240.4±33.35 | 4.27±1.27 | 4.00±0.34 | 5.50±1.09 | 5.55±1.44 | 19.82±5.63 | 21.00±6.22 |  |
| **C5** | **Males** | Cont. | 14.35±1.12 | 26.34±1.96 | | 258.9±25.67 | 5.01±1.04 | 5.01±1.47 | 5.72±1.83 | 5.73±1.07 | 21.90±6.45 | 21.93±8.02 |  |
|  |  | CEH | 14.44±0.85 | 26.50±1.37 | | 260.7±22.28 | 5.07±1.07 | 4.30±0.84 | 5.62±1.90 | 5.61±1.14 | 22.88±7.92 | 21.23±4.65 |  |
|  | **Females** | Cont. | 13.21±0.88 | 24.24±1.86 | | 240.1±24.71 | 4.32±1.08 | 4.32±0.68 | 5.54±1.86 | 5.49±0.67 | 21.20±6.28 | 20.29±6.55 |  |
|  |  | CEH | 13.00±0.96 | 24.43±1.45 | | 242.3±24.69 | 4.23±0.97 | 3.77±1.77 | 5.50±0.95 | 5.50±1.49 | 20.10±8.57 | 20.76±7.29 |  |
| **C6** | **Males** | Cont. | 14.58±0.78 | 26.02±1.26 | | 255.2±29.09 | 4.50±0.95 | 4.50±0.83 | 5.08±0.82 | 5.51±1.39 | 20.98±9.03 | 18.33±6.20 |  |
|  |  | CEH | 14.76±0.88 | 26.26±1.92 | | 259.4±35.20 | 4.62±0.80 | 4.10±1.57 | 5.00±0.99 | 5.46±0.95 | 22.30±9.71 | 18.83±8.75 |  |
|  | **Females** | Cont. | 13.97±0.92 | 24.11±1.99 | | 240.1±30.84 | 3.80±1.16 | 3.80±0.46 | 4.88±0.47 | 5.36±1.28 | 20.40±8.08 | 18.32±7.46 |  |
|  |  | CEH | 13.52±0.67 | 24.55±1.81 | | 222.0±25.01 | 3.88±0.46 | 3.88±0.35 | 4.95±1.74 | 5.39±1.35 | 20.09±6.75 | 18.16±5.36 |  |
| **C7** | **Males** | Cont. | 14.61±1.57 | 26.32±2.26 | | 245.0±53.27 | 4.04±1.02 | 4.00±1.76 | 4.72±1.36 | 5.22±0.83 | 16.81±6.10 | 16.66±7.91 |  |
|  |  | CEH | 14.78±0.93 | 26.43±1.76 | | 253.0± 21.65 | 4.10±1.28 | 4.20±1.53 | 4.60±0.74 | 5.00±1.61 | 16.00±7.97 | 17.19±9.11 |  |
|  | **Females** | Cont. | 14.32±1.49 | 24.25±1.72 | | 240.2±32.62 | 3.40±0.90 | 3.40±1.56 | 4.20±1.66 | 4.68±1.57 | 13.70±7.82 | 14.01±8.33 |  |
|  |  | CEH | 13.90±1.22 | 24.61±1.36 | | 207.0±26.90 | 3.50±1.78 | 3.70±0.92 | 4.33±1.56 | 4.62±1.08 | 14.10±5.13 | 13.82±6.19 |  |
| **T1** | **Males** | Cont. | 15.15±1.30 | 22.34±0.88 | | 220.5±23.10 | 6.87±1.89 | 6.78±0.14 | 6.74±0.79 | 6.62±1.75 | 33.36±9.27 | 34.82±8.22 |  |
|  |  | CEH | 15.04±1.33 | 22.71±1.83 | | 259.4±22.20 | 6.86±0.96 | 6.65±1.37 | 6.72±0.38 | 6.57±1.66 | - | - |  |
|  | **Females** | Cont. | 14.22±1.27 | 21.69±1.49 | | 222.0±29.50 | 6.37±1.82 | 6.23±1.75 | 6.49±0.44 | 6.30±0.89 | - | - |  |
|  |  | CEH | 14.12±1.63 | 21.15±1.30 | | 210.3±25.30 | 6.16±1.53 | 6.32±1.27 | 6.41±1.28 | 6.10±1.09 | - | - |  |
| CEH = cervicogenic headache group (n=40); Cont. = Control group (n=40); SCL = spinal canal length; SCW = spinal canal width; SCA = spinal canal area; RTFL = right transverse foramen length; LTFL = left transverse foramen length; RTFW = right transverse foramen width; LTFW = left transverse foramen width; RTFA = right transverse foramen area; LTFA = left transverse foramen area; C1-C7 = cervical vertebrae, T1 = thoracic vertebra; mm = millimeters | | | | | | | | | | | | | |
